# Loss of PTEN expression in breast cancer: association with clinicopathological characteristics and prognosis

**DOI:** 10.18632/oncotarget.16761

**Published:** 2017-03-31

**Authors:** Shuting Li, Yanwei Shen, Mengying Wang, Jiao Yang, Meng Lv, Pan Li, Zheling Chen, Jin Yang

**Affiliations:** ^1^ Department of Medical Oncology, The First Affiliated Hospital of Xi'an Jiaotong University, Xi'an, Shaanxi, P.R. China; ^2^ Institute of Endemic Diseases, Xi'an Jiaotong University Health Science Center, Xi'an, Shaanxi, P.R. China; ^3^ Key Laboratory of Environment and Genes Related to Diseases, Xi'an Jiaotong University Health Science Center, Xi'an, Shaanxi, P.R. China

**Keywords:** PTEN, breast cancer, prognosis, meta-analysis

## Abstract

Various studies have evaluated the significance of PTEN (phosphatase and tensin homolog deleted from chromosome 10) expression in breast cancer, but their results remain controversial. We conducted a meta-analysis to evaluate the associations of PTEN expression with clinicopathological characteristics and prognosis in breast cancer. PubMed, Embase, Web of Science, and China National Knowledge Infrastructure were searched to identify relevant publications. The associations between PTEN expression and clinicopathological parameters, disease-free survival (DFS), and overall survival (OS) were then assessed via meta-analyses of odds ratio (ORs) and hazard ratio (HRs) with 95% confidence intervals (CIs). Based on 27 studies involving 10,231 patients, the pooled results revealed that PTEN loss was significantly more common in breast cancer than in normal tissues (OR = 12.15, 95% CI = 6.48–22.79, *P* < 0.00001) and that PTEN loss had clear associations with larger tumor size (> 2 cm, OR = 0.62, 95% CI = 0.48–0.82, *P* *=* 0.0006), lymph node metastasis(OR = 0.61, 95% CI = 0.45–0.82, *P* = 0.0001), later TNM stage(stage III–IV, OR = 0.55, 95% CI = 0.35–0.86, *P* *=* 0.009), poor differentiation(OR = 0.37, 95% CI = 0.24–0.59, *P* < 0.0001), and the highly aggressive triple-negative phenotype (OR = 1.62, 95% CI = 1.23–2.12, *P* = 0.0005). Moreover, patients with PTEN loss exhibited significantly worse DFS and OS(HR = 1.63, 95% CI = 1.04–2.22, *P* < 0.00001; HR = 1.41, 95% CI = 1.08–1.73, *P* < 0.0001; respectively). In conclusion, PTEN loss might predict more aggressive behavior and worse outcomes in patients with breast cancer.

## INTRODUCTION

Breast cancer is the most common malignancy among women in both developing and developed countries, accounting for about one fifth of new cancer cases in women [1]. The outcomes of breast cancer have improved substantially during the past few decades as a result of recent advancements in the understanding of breast cancer biology and the development of new protocols for individual treatments. However, breast cancer still remains the leading cause of death in women worldwide [2]. Therefore, it is important to identify potential biomarkers that could be used to screen high-risk patients and predict breast cancer prognosis in conjunction with classical pathological parameters.

The phosphatase and tensin homolog deleted from chromosome 10 (PTEN) tumor suppressor is a negative regulator of PI3K/AKT signaling, directly and indirectly affecting cell survival, proliferation, and apoptosis [3]. PTEN dephosphorylates the 3′ end of the triphosphate PIP3 in the inositol ring, resulting in the biphosphate PIP2, which inhibits AKT activation and downstream signaling processes that depend on AKT for activation. Inactivation of PTEN, and thus lack of inhibition of the AKT-dependent processes, has been associated with tumorigenesis in multiple human cancers, including breast cancer [4].

In breast cancer, the frequency and relevance of PTEN alterations have not been elucidated completely. In the prior literature, it has been reported that PTEN deletions or reduced expression are present in 4% to 63% of breast cancer cases [5, 6]. Recently, several groups have analyzed PTEN expression patterns and the correlations of PTEN expression with clinicopathological features and clinical outcomes. However, the results of these studies have been conflicting and controversial. Some studies have suggested that there is an association between PTEN inactivation and poor prognosis in breast cancer patients [7, 8], whereas this could not be confirmed in other studies [9, 10]. Therefore, we decided that a comprehensive investigation would be useful to clarify the expression status and prognostic significance of PTEN. Accordingly, we performed a meta-analysis, incorporating all of the currently available evidence to evaluate the relationships between PTEN loss, clinicopathological parameters, and clinical outcomes of breast cancer.

## RESULTS

### Study searches and population characteristics

**Figure 1:**
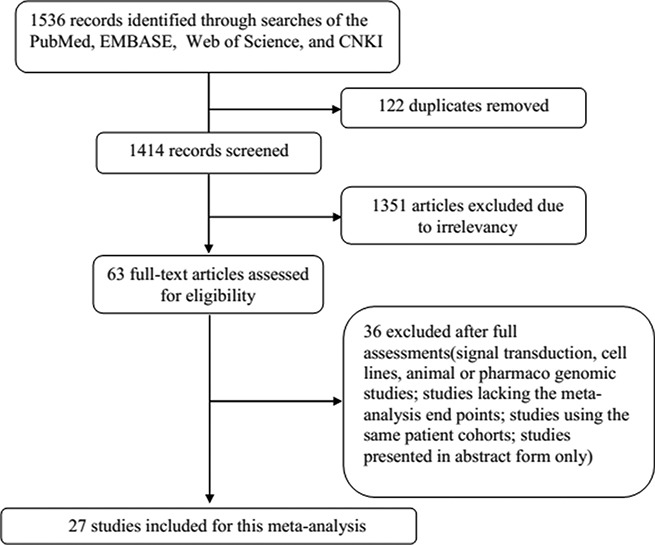
Flow diagram of the details of this study

Overall, a total of 1536 potentially relevant records were identified using the search strategy mentioned above. However, 122 duplicates were removed by EndNote and 1351 studies were excluded by reading their titles and abstracts. In a preliminary analysis that included detailed sorting and reading, 63 studies were considered for inclusion in the meta-analysis. However, 36 of these studies were excluded because they reported animal or cell experiments, lacked clinical specimens, or were presented in abstract form only. Ultimately, 27 studies [5, 6, 8, 11–34] were included in the final analysis. A flow diagram of the article retrieval and selection process is provided in Figure 1.

The detailed characteristics of all eligible studies are shown in Table 1. Specifically, all 27 candidate studies assessed relationships between PTEN loss and clinicopathological parameters of breast cancer patients, while 9 publications estimated associations between PTEN loss and breast cancer prognosis. Together, these 27 studies included 10,231 patients from China, the USA, theUK, Brazil, Italy, Germany, Denmark, Iran, Japan, Korea, Saudi Arabia, Turkey, the Netherlands, and Poland, with a mean sample size of 379 patients per study (range, 34 to 2364). Immunohistochemistry (IHC) methods were adopted in 25 of the included studies to detect PTEN expression in breast cancer specimens. Among the included articles, the expression of PTEN in tumor cells was most commonly evaluated based on the percentage of positively stained cells or the staining intensity score.

**Table 1:** Summary characteristics of all eligible studies

Study (publication year, country)	Sample size	Age (year)	Histological type	Detection method	Evaluationmethod	Cut-off value for positive PTEN	Staining pattern	Outcome indexes
Bose et al. (2002, USA) [11]	34	NA	Mixed	IHC	SI	> 0	Nuclear/cytoplasm	NA
Capodanno et al. (2009, Italy) [8]	72	55 (34–82)	Mixed	IHC	PP	≥ 10%	Nuclear/cytoplasm	DFS
Wang et al. (2016, China) [12]	296	51 (20–78)	Mixed	IHC	PP	≥ 5%	Membrane/cytoplasm	OS
Lima Lin et al. (2014, Brazil) [13]	104	54 (30–91)	Mixed	IHC	PP	≥ 10%	Nuclear	NA
Lebok et al. (2015, Germany) [14]	1239	63 (26–101)	Mixed	FISH	PP	≥ 60%	NA	OS
Palimaru et al. (2013, Denmark) [15]	175	64 (32–85)	Mixed	RT-PCR	CS	NA	NA	NA
Noh et al. (2008, Korea) [5]	122	NA	Mixed	IHC	SI	> 0	Cytoplasm	NA
Li et al. (2015, China) [16]	291	50 (26–78)	Mixed	IHC	PP	≥ 10%	Nuclear	NA
Golmohammadi et al. (2016, Iran) [17]	100	47 (25–82)	Mixed	IHC	PP	≥ 10%	Nuclear	NA
Cuorvo et al. (2014, Italy) [18]	210	NA	Mixed	IHC	SI	> 0	Nuclear/cytoplasm	NA
Arthur et al. (2014, UK) [19]	96	66 (25–94)	Mixed	IHC	H-score	≥ 100	NA	OS
Beg et al. (2015, Saudi Arabia) [20]	957	NA	Mixed	IHC	H-score	≥ 90	Nuclear/cytoplasm	OS
Inanc et al. (2014, Turkey) [21]	97	47 (27–79)	Mixed	IHC	SI	> 0	Membrane/cytoplasm	DFS
Beelen et al. (2014, Netherlands) [22]	436	NA	Mixed	IHC	SI	> 0	Cytoplasm	NA
Lazaridis et al. (2014, Germany) [34]	997	NA	Mixed	IHC	SI	> 0	Nuclear/cytoplasm	NA
Chung et al. (2004, Korea) [23]	88	55 (36–70)	Mixed	IHC	SI	≥ 2	Cytoplasm	NA
Iqbal et al. (2012, China) [6]	144	53 (28–88)	Mixed	IHC	PP	≥ 10%	Nuclear/cytoplasm	DFS
Szmich et al. (2015, Poland) [24]	78	NA	Mixed	IHC	PP	≥ 15%	Nuclear	NA
Stern et al. (2015, USA) [25]	2364	NA	Mixed	IHC	SI	> 0	Nuclear/cytoplasm	NA
Sueta et al. (2015, Japan) [26]	41	NA	Mixed	IHC	H-score	≥ 60	Nuclear/cytoplasm	NA
Perez et al. (2015, China) [27]	1802	50 (22–80)	Mixed	IHC	SI	≥ 2	Cytoplasm	NA
Chen et al. (2014, China) [28]	130	56 (21–75)	Mixed	IHC	SI	> 0	Cytoplasm	DFS, OS
Tang et al. (2014, China) [31]	68	53 (30–71)	Mixed	IHC	SI	> 0	Nuclear	Mortality
Lu et al. (2006, China) [33]	60	46 (32–75)	Mixed	IHC	PP	≥ 10%	Nuclear	NA
Tian et al. (2008, China) [32]	72	51 (38–73)	Mixed	IHC	SI	> 0	Nuclear/cytoplasm	NA
Huang et al. (2012, China) [29]	90	NA	Mixed	IHC	H-score	≥ 30	Cytoplasm	NA
Fang et al. (2013, China) [30]	68	52 (31–72)	Mixed	IHC	H-score	≥ 30	Nuclear/cytoplasm	DFS

Notes: Age is given as mean (range). H-score = SI (staining intensity) × PP (percentage of positive cells). SI was determined as: 0, negative; 1, weak; 2, moderate; and 3, strong. PP was defined as: 0, negative; 1–100, 1–100% positive cells.

Abbreviations: NA, not available; IHC, immunohistochemistry; FISH, fluorescent *in situ* hybridization; RT-PCR, real-time polymerase chain reaction; SI, staining intensity; PP, percentage of positive cells; CS, complex scoring; OS, overall survival; DFS, disease-free survival.

### Quality assessment

For each of the 27 eligible studies, validity was evaluated using the Newcastle–Ottawa Scale, as described previously. Studies that fulfilled six or more of the eight criteria were regarded as higher quality studies. Overall, the mean Newcastle–Ottawa Scale score was 6.5 (range, 5 to 8). The scores of the included studies are shown in Supplementary Table 1.

### PTEN loss correlates with breast cancer development

**Figure 2:**
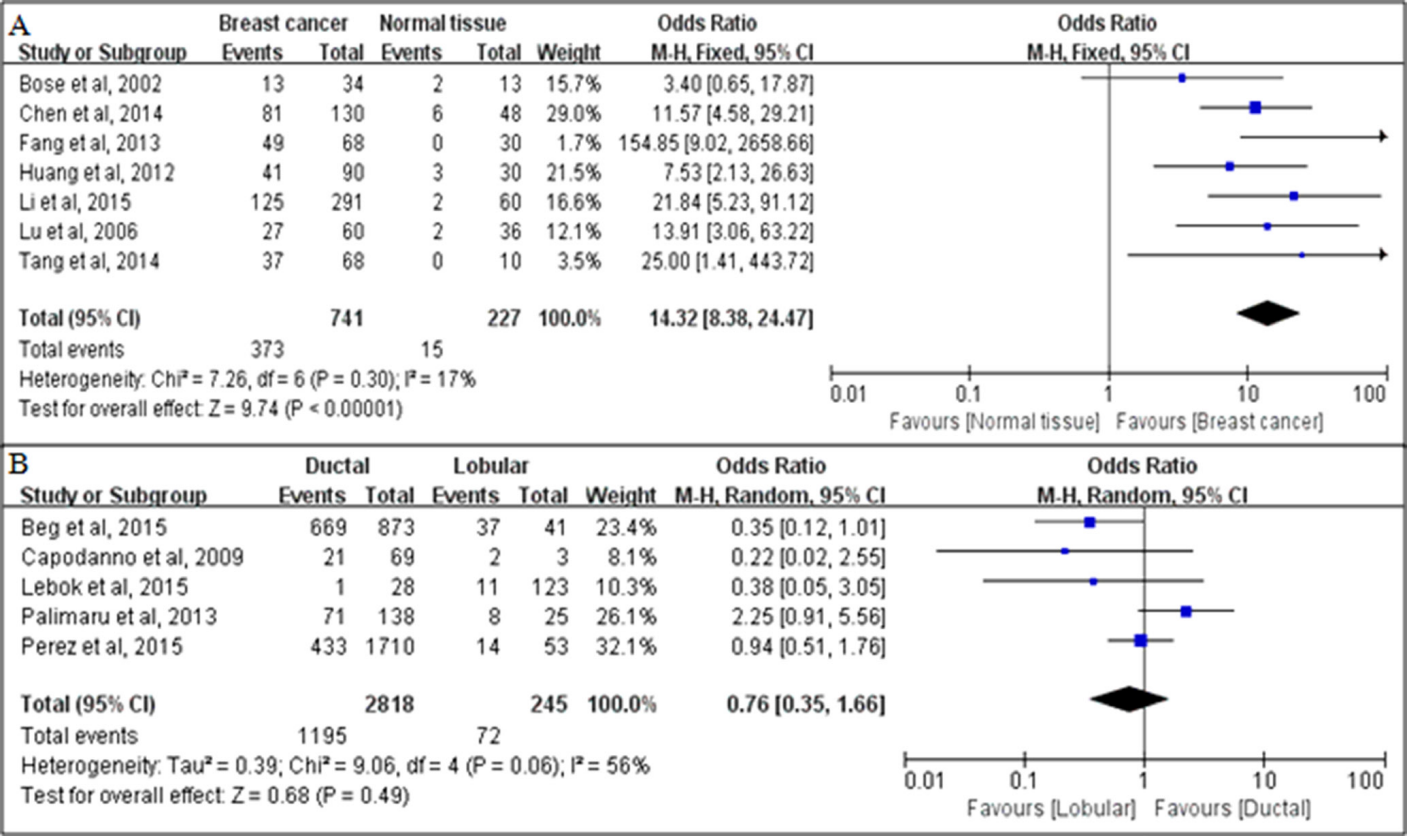
Associations between PTEN loss and breast cancer development, as evaluated in terms of odds ratios (ORs) (**A**) Associations between PTEN loss and risks of breast cancer, compared with normal breast tissues; (**B**) Associations between PTEN loss and the histological type of breast cancer (ductal carcinoma versus lobular carcinoma).

Our meta-analysis incorporated a total of eight studies [11, 16, 28–31, 33] that compared the PTEN loss rate in breast cancer tissues and matched normal tissues, including 741 breast cancer tissue samples and 227 normal samples. The heterogeneity test showed a *I*^2^ value of 17% (*P* = 0.30), and we therefore used a fixed-effects model. The pooled OR was 14.32 (95% CI = 8.38–24.47, *P <* 0.00001), indicating that the PTEN loss rate in breast cancer was significantly higher than that in normal tissues (Figure 2A). Further, five studies [8, 14, 15, 20, 27] investigated the relationship between PTEN loss and the histological type of breast cancer. The heterogeneity test indicated that a random-effects model should be selected (*I*^2^ = 56%, *P* = 0.06). However, in the pooled results, the PTEN loss rate did not differ significantly between ductal carcinoma and lobular carcinoma (OR = 0.76; 95% CI = 0.35–1.66; *P* = 0.49) (Figure 2B).

### PTEN loss correlates with breast cancer progression

**Figure 3:**
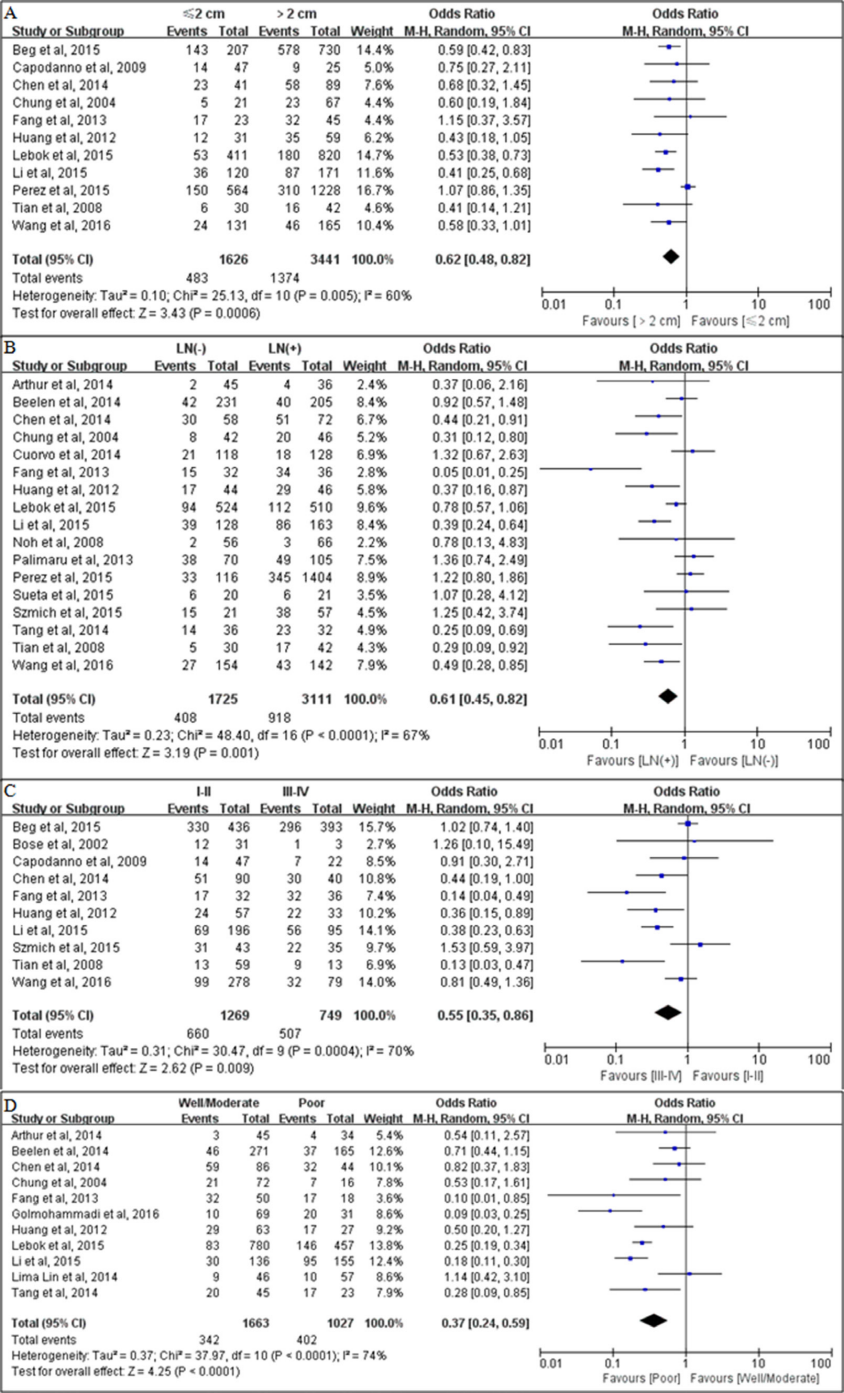
Associations between PTEN loss and clinicopathological parameters (**A**) Associations between PTEN loss and tumor size; (**B**) Associations between PTEN loss and lymph node metastasis status; (**C**) Associations between PTEN loss and TNM stage; (**D**) Associations between PTEN loss and tumor differentiation.

A total of 11 studies [8, 12, 14, 16, 20, 23, 27–30, 32] analyzed the relationship between PTEN loss and tumor size. Considering the presence of heterogeneity in the study findings (*P* = 0.005, *I*^2^ = 60%), we selected a random-effects model method for further analysis. The pooled OR was 0.62 (95% CI = 0.48–0.82, *P* = 0.0006; Figure 3A), indicating that PTEN loss was significantly associated with larger tumor size (> 2 cm). Twelve studies [5, 12, 14–16, 18, 19, 22–24, 26–32] investigated the relationship between PTEN loss and lymph node metastasis status. The pooled results suggested that PTEN loss was significantly associated with the presence of lymph node metastasis (pooled OR = 0.61, 95% CI = 0.45–0.82, *P* = 0.0001 using a random-effects model; Figure 3B). Ten studies [8, 11, 12, 16, 20, 24, 28–30, 32] evaluated the relationship between PTEN loss and TNM stage. A statistically significant association was observed between PTEN loss and TNM stage III–IV, with a pooled OR of 0.55 (95% CI = 0.35–0.86, *P* = 0.009 using a random-effects model; Figure 3C).In addition, a clear association was observed between PTEN loss and poorly differentiated breast cancer (OR = 0.37, 95% CI = 0.24–0.59; *P <* 0.0001 using a random-effects model; Figure 3D).

### PTEN loss according to molecular subtype

**Figure 4:**
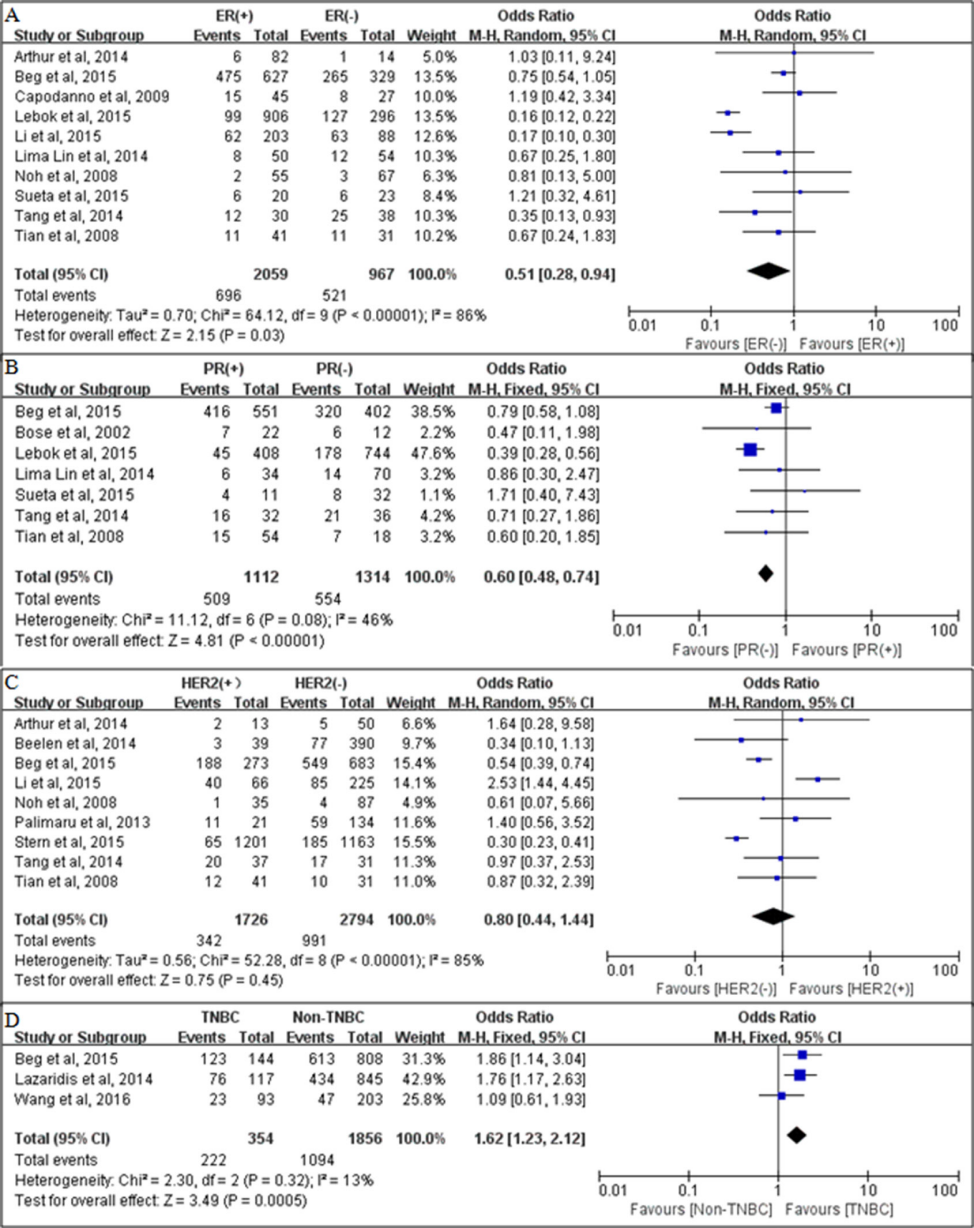
Associations between PTEN loss and molecular subtype of breast cancer The relationships between PTEN loss and (**A**) The relationships between PTEN loss and (**B**). progesterone receptor (PR) status; (**C**) human epidermal growth factor receptor 2 (HER2) status; and (**D**) triple-negative breast cancer (TNBC).

We also analyzed the associations between PTEN loss and the molecular subtype of breast cancer. PTEN loss was significantly associated with negative estrogen receptor (ER) expression (pooled OR = 0.51, 95% CI = 0.28–0.94, *P* = 0.03 using a random-effects model; Figure 4A) and negative progesterone receptor (PR) expression (pooled OR = 0.64, 95% CI = 0.44–0.93, *P* = 0.02 using a random-effects model; Figure 4B). There was no significant relationship between PTEN loss and human epidermal growth factor receptor 2 (HER2) status (pooled OR = 0.80, 95% CI = 0.44–1.44, *P* = 0.45 using a random-effects model; Figure 4C). However, PTEN loss was found to be significantly associated with the biologically aggressive triple-negative phenotype (pooled OR = 1.62, 95% CI = 1.23–2.12, *P* = 0.0005 using a fixed-effects model; Figure 4D).

### Associations between PTEN loss and survival

**Figure 5:**
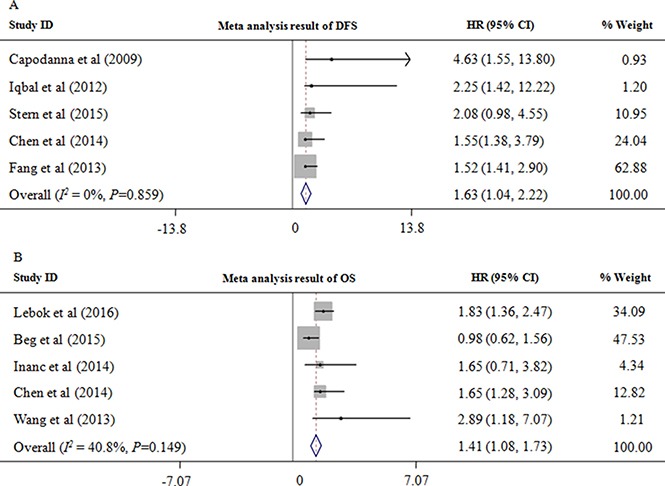
PTEN loss is associated with a poor prognosis (**A**) Associations between PTEN loss and disease-free survival (DFS) for patients with breast cancer. (**B**) Associations between PTEN loss and overall survival (OS) for patients with breast cancer. HR, hazard ratio.

We evaluated the associations between PTEN loss and the survival outcomes of breast cancer patients. A total of five studies [6, 8, 25, 28, 30] examined the association between PTEN loss and DFS. The combined HR was calculated using a fixed-effects model (*P* = 0.859, *I*^2^ = 0%), which showed that PTEN loss was associated with significantly shorter DFS (HR =1.63, 95% CI = 1.04–2.22, *P <* 0.00001; Figure 5A). Moreover, five studies [12, 14, 20, 21, 28] evaluated the association between PTEN loss and OS. Using a random-effects model, the pooled estimate demonstrated a significant relationship between PTEN loss and poorer OS (HR = 1.41, 95% CI = 1.08–1.73, *P <* 0.0001; Figure 5B), indicating that PTEN loss predicted worse prognosis in breast cancer patients.

### Sensitivity analysis

**Figure 6:**
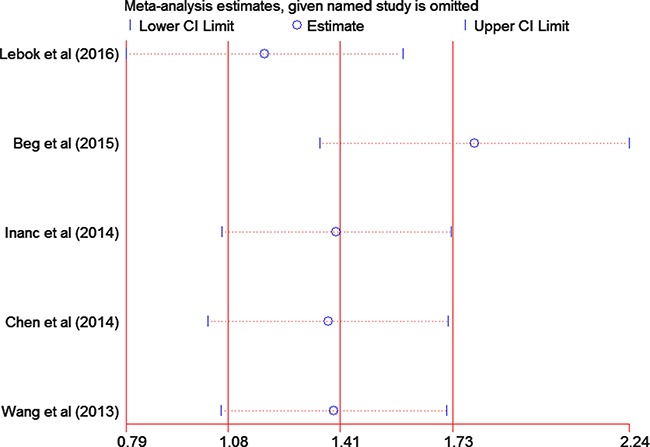
Sensitivity analysis of the summary hazard ratio for overall survival The results were computed by omitting each study in turn. Meta-analysis random-effects estimates (exponential form) were used. The two ends of the dotted lines represent the 95% confidence intervals.

Significant heterogeneity was observed among the included studies of OS (*I*^2^ = 40.8%). As shown in Figure 6, the study conducted by Beg et al. [20] showed OS results that were substantially different from those of the remaining studies, which had probably contributed to the heterogeneity. After excluding this study, a significant association continued to be observed between PTEN loss and OS (HR=1.80; 95% CI = 1.35–2.24; *P <* 0.00001) without any evidence of heterogeneity among the remaining studies (*P* = 0.877, *I*^2^ = 0%).

### Publication bias

**Figure 7:**
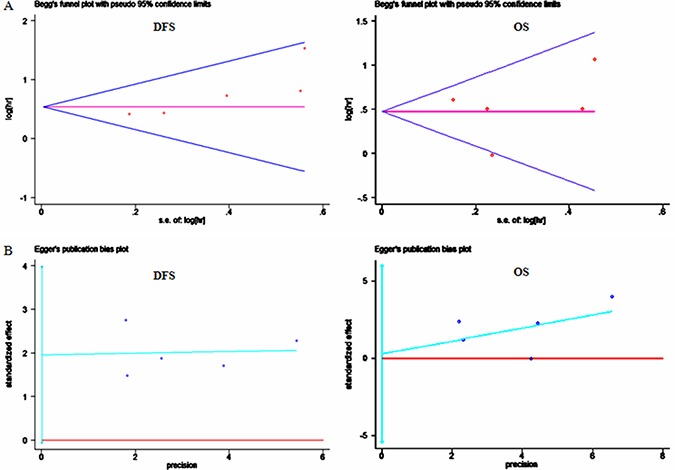
Analyses of publication bias for the relationships between PTEN loss and survival (**A**) Begg's funnel plots of publication bias tests for the overall merged analyses of disease-free survival (DFS) and overall survival (OS); (**B**) Egger's test of effect sizes for the overall merged analyses of DFS and OS. Each point represents a separate study.

To gauge the stability of overall estimates, Begg's funnel plot and Egger's test were used to investigate potential publication bias among the included studies. The Begg's funnel plots appeared to be symmetric, showing no evidence of substantial publication bias for the pooled DFS or OS results (Figure 7A), which was further supported by the results of the Egger's tests (*t* = 3.10, *P* = 0.054; *t* = 0.14, *P* = 0.894; respectively) (Figure 7B).

## DISCUSSION

Despite the remarkable advancements in treatment that have been provided by personalized therapy, breast cancer remains the most common malignancy in women, making it a major public health challenge. New reliable prognostic markers are needed to identify those patients who are at high risk of disease recurrence, and who would therefore benefit from more aggressive adjuvant therapy and/or closer follow-up. To date, several meta-analyses have demonstrated that various biomarkers may be associated with the survival of patients with breast cancer, including p27 [35], vascular endothelial growth factor (VEGF) [36], Cyclooxygenase (COX-2) [37], B cell lymphoma 2 protein (BCL-2) [38], and cyclin D1 [39].

The *PTEN* gene at 10q23 encodes a lipid phosphatase that functions as a direct antagonist of phosphatidylinositol 3-kinase and is involved in the regulation of the AKT pathway. Inactivation of *PTEN* leads to constitutively activated levels of AKT, thus promoting cell growth, proliferation, survival, and migration through multiple downstream effectors [40]. Previous studies have shown that *PTEN* is aberrantly downregulated and acts as a tumor suppressor in several types of cancer, and that PTEN loss is an unfavorable factor in predicting the outcomes of cancer [41], colorectal cancer [42], non-small cell lung cancer [43], diffuse large B-cell lymphoma [44], mesothelioma [45], and prostate cancer [46]. However, the association between PTEN loss and prognosis has been controversial in patients with breast cancer. We thought that a meta-analysis could help to clarify this issue.

To the best of our knowledge, the current study is the first meta-analysis to systematically evaluate the associations of PTEN loss with the clinicopathological parameters and prognosis of breast cancer. Our results showed that the rate of PTEN loss was significantly higher in breast cancer tissues than in matched normal tissues, which suggests that PTEN might play an important role in the development of breast cancer. Furthermore, the pooled findings from our meta-analyses of 27 studies (including 10,231 cases) provide compelling evidence of a significant correlation between PTEN loss and aggressive behavior of breast cancer, including larger tumor size, lymph node metastasis, later TNM stage, and poor differentiation. Considering that breast cancer is a heterogeneous disease, we also sought to investigate the associations between PTEN loss and the molecular subtype of breast cancer. The pooled findings showed that PTEN loss was significantly associated with negative ER expression, negative PR expression, and the triple-negative phenotype of breast cancer. All of these results support the hypothesis that PTEN plays a tumor suppressor role in breast cancer, and the idea that PTEN loss is involved in the initiation and malignant progression.

In the present meta-analysis, we also assessed the associations of PTEN loss with the OS and DFS of patients with breast cancer. The pooled findings of the included studies indicated that PTEN loss was significantly associated with poor OS and DFS. Although there was significant heterogeneity among the included studies of OS, a sensitivity analysis showed that the study by Beg et al. [20] seemed to be the major source of this heterogeneity. After removing study by Beg et al. the heterogeneity disappeared among the remaining studies. Neither Begg's nor Egger's tests revealed any significant publication bias for DFS or OS, demonstrating that our data are robust and reliable. Taking all of our study's findings into consideration, we believe that PTEN is a promising prognostic marker that could provide helpful prognostic information during the clinical decision-making process for breast cancer treatments.

Although we attempted to make our study as comprehensive as possible, it has several limitations First, HRs were sometimes unavailable in the included studies, and were therefore obtained from indirect calculations and estimates based on survival data or Kaplan–Meier curves, which may have compromised the precision of the data included in our meta-analyses. Second, the cutoff values that were used to distinguish between positive and negative immunohistochemical expression varied across the included studies, which might have introduced heterogeneity into the overall results. Third, most of the eligible studies failed to provide data regarding progression-free survival or recurrence-free survival, and our meta-analyses were therefore limited to DFS and OS. Further, scientists may be less likely to report negative results for a prognostic biomarker, leading to publication bias. Additionally, to provide more reliable results, there is a need for well-designed studies that use a unified definition in assessments of protein expression, include uniform cases, and involve larger sample sizes.

In conclusion, although the current meta-analysis is subject to some limitations, its results identify PTEN loss as a frequent event in breast cancer that is closely associated with progression and poor prognosis. PTEN may be a promising, useful biomarker for predicting clinical outcomes in women with breast cancer.

## MATERIALS AND METHODS

### Search strategy

This meta-analysis followed the Preferred Reporting Items for Systematic Reviews and Meta-Analyses (PRISMA) statement proposed by the Cochrane Collaboration [47]. To identify all articles that investigated associations between PTEN expression and breast cancer, we searched for published literature in the electronic databases PubMed, EMBASE, China National Knowledge Infrastructure and Web of Science. Our searches were limited to articles that had been published in English or Chinese before October 2016. The searches used the following terms: (“PTEN” OR “MMAC-1” OR “phosphatase tensin homologue deleted on chromosome 10”) AND (breast carcinoma OR breast cancer) AND (prognosis OR prognostic OR outcome OR mortality OR survival). In addition, the bibliographies of articles and the supplemental materials associated with studies were examined manually to identify any other relevant publications.

### Inclusion and exclusion criteria

To be included in the meta-analysis, all studies had to meet the following inclusion criteria: 1) the study focused on breast cancer patients with pathologically confirmed disease; 2) the detection method used immunohistochemistry(IHC), fluorescent *in situ* hybridization (FISH), or real-time polymerase chain reaction (RT-PCR), with findings that were analyzed quantitatively; 3) the main outcomes of interest were associations between PTEN expression and overall survival (OS) and/or other clinicopathological parameters; 4) the studies that aimed to explore associations between PTEN expression and survival status must have provided sufficient data to estimate hazard ratio (HR) for overall survival (OS) or disease-free survival (DFS), as well as 95% confidence intervals (CIs); 5) the sample size was more than 30 cases; 6) if there were multiple articles on the same or overlapping cohorts, only the most complete and/or recently published article was included; and 7) the original articles were written in English or Chinese.

Further, studies that met the following criteria were excluded: 1) abstracts, letters to the editor, reviews, comments, duplicated studies, and articles published in books; 2) articles which failed to present the cutoff value that was used to define PTEN positivity; 3) studies had duplicate data or lacked key information that was needed to estimate an OR or HR and 95% CI; and 4) studies that were based on animal or human cell lines.

Two investigators (LST and SYW) were independently involved in search and identification. Inclusion and exclusion decisions were reached by two investigators after evaluating the manuscripts. If views diverged, the differences were resolved through iteration, discussion, and consensus between the two investigators or consulting with a third investigators (LM).

### Data extraction

For the studies included in our meta-analysis, data extraction was performed independently by two investigators (LST and SYW). The form used for data extraction documented the most relevant items, including the name of the first author and publication year, country, study period, study population characteristics (patient numbers, ages, and sex), PTEN assessment method, cutoff value for positive PTEN expression, staining pattern, outcome indexes, and HRs and associated 95% CIs for DFS and OS. Any remaining uncertainties were addressed by joint inspection of the papers and discussion. All relevant text, tables, and figures were reviewed for data extraction. We contacted the authors of the eligible studies for information on missing data.

### Qualitative assessment

Two investigators (YJ and CZL) independently assessed the methodological quality of each study using the Newcastle–Ottawa Quality Assessment Scale(http://www.ohri.ca/programs/clinical_epidemiology/oxford.asp; Accessed July 20, 2012), which is recommended by the Cochrane Non-Randomized Studies Methods Working Group. This scale uses a star system(a score of 0–9) to indicate the quality of each study based on its patient population and selection, study comparability, follow-up, and outcome of interest [48]. Studies were considered to be of high quality if they received six or more stars. Any disagreement was resolved by consensus.

### Statistical analysis

The guidelines recommended by the Meta-Analysis of Observational Studies in Epidemiology (MOOSE) group were applied during the statistical analyses. To aggregate prognostic results in a quantitative manner, HRs and their 95% CIs were combined to obtain an overall measure of effect. When these data were not directly provided in the eligible studies, we reconstruct the HR estimate and its variance from Kaplan–Meier survival curves using Engauge Digitizer version 4.1 (free software downloaded from http://sourceforge.net). To assess the relationships between PTEN loss and clinicopathologic features of breast cancer, estimated ORs and 95% CIs were combined to obtain an overall measure of association. Statistical heterogeneity across the incorporated studies was measured using the *Q*-test and inconsistency (*I*^2^) test. The significance of the pooled OR was determined using a *Z*-test (*P <* 0.05 was considered statistically significant). Both fixed- and random-effects models could be used in the absence of heterogeneity, but random-effects models were regarded as being more appropriate when heterogeneity was present. To assess the degree of potential publication bias both graphically and statistically, funnel plots were created and Egger's tests were performed. Asymmetric plots were interpreted as suggesting the possible existence of publication bias, while *P <* 0.05 was interpreted as indicating statistically significant publication bias. All statistical analyses were performed in Stata 12.0 (Stata Corporation, College Station, TX, USA) and Review Manager 5.2 (Cochrane Collaboration, London, UK).

## SUPPLEMENTARY MATERIALS TABLE


