# Supplementary material for: Loss of PTEN expression in breast cancer: association with clinicopathological characteristics and prognosis
Source: Oncotarget. 2017 Mar 31;8(19):32043–54. doi: 10.18632/oncotarget.16761 (PMC5458267; doi:10.18632/oncotarget.16761)
Supplement: Supplementary file 1 [file oncotarget-08-32043-s001.pdf]

Loss of PTEN expression in breast cancer: association with clinicopathological characteristics and prognosis

Supplementary Materials

Supplementary Table 1: Quality of the included studies based on the newcastle-ottawa scale

Studies	Selection				Comparability	Outcome			Total core
	Representativeness of the exposed cohort	Selection of the non exposed cohort	Assessment of exposure	Outcome not present at start of study		Assessment of outcome	Follow-up long enough for outcomes	Adequacy of follow up	
Bose et al. (2002, USA) [11]	1	1	1	1	1	1	1	1	8
Capodanno et al. (2009, Italy) [8]	1	1	1	1	1	0	1	0	6
Wang et al. (2016, China) [12]	1	1	1	1	0	1	1	0	6
Lima Lin et al. (2014, Brazil) [13]	1	1	1	1	1	1	1	0	7
Lebok et al. (2015, Germany) [14]	1	1	1	1	1	1	1	0	7
Palimaru et al. (2013, Denmark) [15]	1	1	1	1	0	1	1	0	6
Noh et al. (2008, Korea) [5]	1	1	1	1	1	1	0	0	6
Li et al. (2015, China) [16]	1	1	1	1	0	1	0	0	5
Golmohammadi et al. (2016, Iran) [17]	1	1	1	1	1	1	0	0	6
Cuorvo et al. (2014, Italy) [18]	1	1	1	1	1	1	1	0	7
Arthur et al. (2014, UK) [19]	1	1	1	1	1	1	1	0	7
Beg et al. (2015, Saudi Arabia)[20]	1	1	1	1	1	1	1	0	7
Inanc et al. (2014, Turkey) [21]	1	1	1	1	1	1	0	0	6
Beelen et al. (2014, Netherlands) [22]	1	1	1	1	1	1	1	0	7
Lazaridis et al. (2014, Germany) [34]	1	1	1	1	1	1	1	0	7
Chung et al. (2004, Korea) [23]	1	1	1	1	1	1	0	1	7
Iqbalet al. (2012, China) [6]	1	1	1	1	1	1	1	1	8
Szmichet al. (2015, Poland) [24]	1	1	1	1	0	1	1	0	6
Stern et al. (2015, USA) [25]	1	1	1	1	1	1	1	1	8
Sueta et al. (2015, Japan) [26]	1	1	1	1	1	1	1	0	7
Perez et al. (2015, China) [27]	1	1	1	1	1	0	1	1	7
Chen et al. (2014, China) [28]	1	1	1	1	1	1	1	0	7
Tang et al. (2014, China) [31]	1	1	1	1	0	1	0	1	6
Lu et al. (2006, China) [33]	1	1	1	1	1	0	1	0	6
Tian et al. (2008, China) [32]	1	1	1	1	1	1	0	0	6
Huang et al. (2012, China) [29]	1	1	1	1	1	1	0	0	7
Fang et al. (2013, China) [30]	1	1	1	1	1	1	1	0	7
